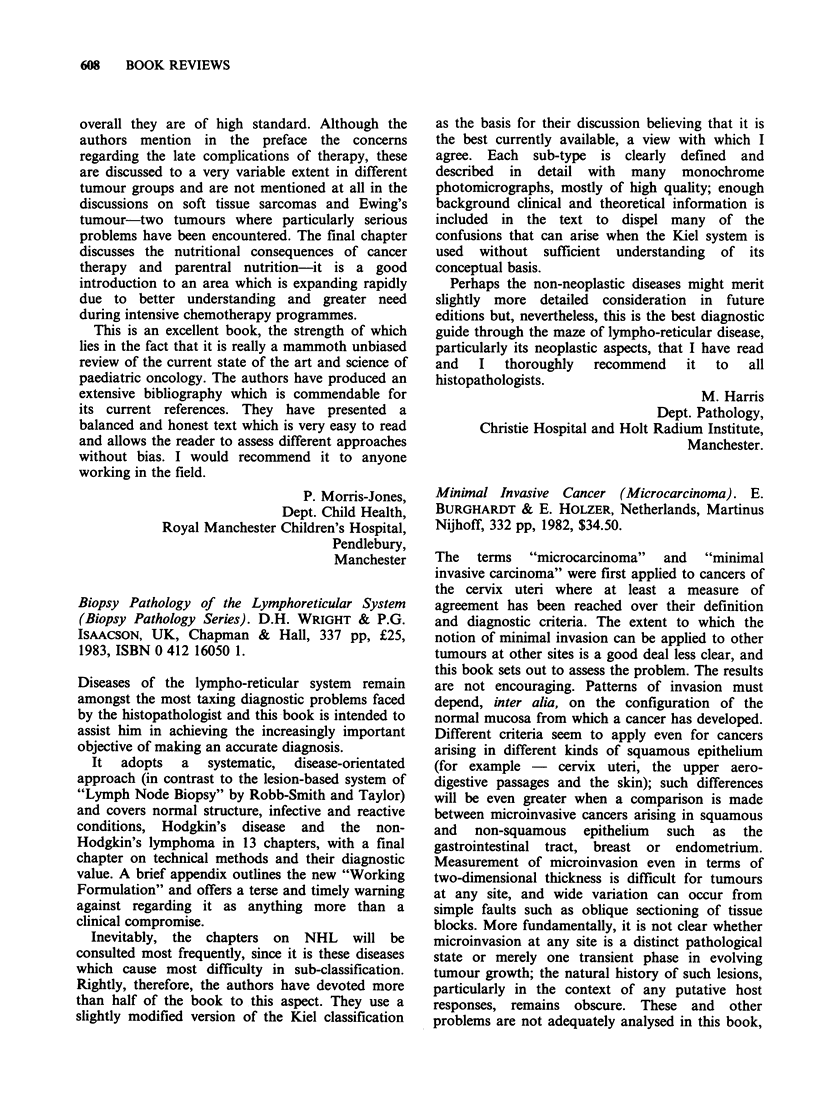# Biopsy Pathology of the Lymphoreticular System (Biopsy Pathology Series)

**Published:** 1983-10

**Authors:** M. Harris


*Biopsy Pathology of the Lymphoreticular System (Biopsy Pathology Series).* D.H. WRIGHT & P.G. ISAACSON, UK, Chapman & Hall, 337 pp, £25, 1983, ISBN 0 412 16050 1.

Diseases of the lympho-reticular system remain amongst the most taxing diagnostic problems faced by the histopathologist and this book is intended to assist him in achieving the increasingly important objective of making an accurate diagnosis.

It adopts a systematic, disease-orientated approach (in contrast to the lesion-based system of "Lymph Node Biopsy" by Robb-Smith and Taylor) and covers normal structure, infective and reactive conditions, Hodgkin's disease and the non-Hodgkin's lymphoma in 13 chapters, with a final chapter on technical methods and their diagnostic value. A brief appendix outlines the new "Working Formulation" and offers a terse and timely warning against regarding it as anything more than a clinical compromise.

Inevitably, the chapters on NHL will be consulted most frequently, since it is these diseases which cause most difficulty in sub-classification. Rightly, therefore, the authors have devoted more than half of the book to this aspect. They use a slightly modified version of the Kiel classification as the basis for their discussion believing that it is the best currently available, a view with which I agree. Each sub-type is clearly defined and described in detail with many monochrome photomicrographs, mostly of high quality; enough background clinical and theoretical information is included in the text to dispel many of the confusions that can arise when the Kiel system is used without sufficient understanding of its conceptual basis.

Perhaps the non-neoplastic diseases might merit slightly more detailed consideration in future editions but, nevertheless, this is the best diagnostic guide through the maze of lympho-reticular disease, particularly its neoplastic aspects, that I have read and I thoroughly recommend it to all histopathologists.

M. Harris
Dept. Pathology,
Christie Hospital and Holt Radium Institute,
Manchester.